# Epidemiology and risk factors of hypovitaminosis D in a cohort of internationally adopted children: a retrospective study

**DOI:** 10.1186/s13052-018-0527-4

**Published:** 2018-07-27

**Authors:** Gilda Salerno, Manuela Ceccarelli, Chiara de Waure, Marianna D’Andrea, Danilo Buonsenso, Valerio Faccia, Davide Pata, Piero Valentini

**Affiliations:** 1 Department of Woman and Child Health, “A. Gemelli” University Hospital Foundation, Unit of Pediatrics, Catholic University of Sacred Heart, Largo Agostino Gemelli, 8, 00168 Rome, Italy; 2 Department of Clinical and Experimental Medicine, Unit of Infectious Diseases, University of Messina, Via Consolare Valeria, 1, 98125 Messina, Italy; 3 Public Health Institute, Catholic University of Sacred Heart, Largo Agostino Gemelli, 8, 00168 Rome, Italy; 4 Department of Gynecologic, Pediatric and Neonatologic Sciences, “Sant’Andrea” University Hospital, Unit of Pediatrics, University “Sapienza” of Rome, Rome, Italy

**Keywords:** Vitamin D deficiency, Internationally adopted children, Parathormone, Rickets

## Abstract

**Background:**

Predictors of hypovitaminosis D were extensively studied in the adult population, leading to an approximately complete understanding of them, while there is a lack of studies in the pediatric population, especially in migrant and internationally adopted children.

In this retrospective study, we tried to identify the major laboratory predictors of hypovitaminosis D in a cohort of internationally adopted children.

**Methods:**

Data were extracted from the database of the “Ethnopediatrics Outpatient Clinic” of the “A. Gemelli” Foundation University Hospital in Rome, Italy. Our study included 873 children evaluated from March 2007 to May 2016. Analysis of variance, chi square test, t test and multivariate logistic regression were performed, a “p” value < 0.05 was considered significant, with a confidence interval of 95%.

**Results:**

We did not find any significant correlation between Vitamin D and Calcium, Phosphates or Magnesium levels within the population we examined. Moreover, parathyroid hormone is not a good predictor of Vitamin D Status.

**Conclusions:**

Considering the strong influence Vitamin D status has not only on bone health, but also on general well-being, it is due to perform a vitamin D assessment as soon as possible, especially in internationally adopted children.

**Electronic supplementary material:**

The online version of this article (10.1186/s13052-018-0527-4) contains supplementary material, which is available to authorized users.

## Background

Vitamin D (VD) is an essential nutrient with hormone-like activity, which regulates calcium and bone metabolism. Moreover, VD has been recently associated with immune status, autoimmune disorders, infectious diseases, decreased response to corticosteroids and psychiatric diseases [1–5].

These associations highlighted the need for a definition of VD optimal values, and this is particularly true for the pediatric population, because of the importance of VD for the bone health and the immune status during the tricky phase represented by the growth period.

Serum 25-hydroxy-vitamin D (25(OH)D) concentration is currently the marker of choice, as it is the major circulating form of VD and its value reflects both the amount produced in the skin after sun exposure and that assumed with food [6].

Most studies consider values < 10 ng/ml or < 20 ng/ml as deficiency and values > 20 ng/ml or >  30 ng/ml as sufficiency, with a gray zone between 10 and 20 ng/ml and 20–30 ng/ml, respectively, indicated as insufficiency [7–11]. When 25(OH)D serum concentration falls below 4 ng/ml, especially if associated with hypocalcaemia, hypophosphatemia and increased serum alkaline phosphatase (ALP), the risk of rickets and osteomalacia in children rises exponentially [12].

Relatively high rates of subclinical vitamin D deficiency (VDD) have been reported in otherwise healthy infants, children and adolescents in several studies, especially from low-income countries [13–26].

Limited data on the prevalence of hypovitaminosis D among internationally adopted children, a population which could be considered “at risk” for geographical origin and a housing system not always subdued to an optimal surveillance, are available, and there is no study assessing parathyroid hormone (PTH) levels in these groups. The relationship between serum concentrations of 25(OH)D and PTH has been considered as a possible way to define VD status, since PTH increases when vitamin D and, consequently, calcium decrease. Studies performed in adults, and very few studies in pediatric subjects, have showed conflicting results about this inverse relationship [27].

Therefore, it would be of utmost importance to define VD levels under which PTH begins to increase, and if it could be considered a marker of bone health or immune status.

We hypothesized that the prevalence of biochemical rickets and hyperparathyroidism in a population of internationally adopted children was higher than in other children subpopulations. Hence, we evaluated the prevalence of hypovitaminosis D in internationally adopted children. Moreover, we tested if some of the population characteristics, such as age, BMI, sex, housing solution before adoption, season and month of observation, country of origin, could be considered risk factors for hypovitaminosis D in this particular cohort, aiming to find a model applicable to all our patients.

In order to define the prevalence of biochemical rickets, we considered the relationship existing between Vitamin D levels and serum calcium, serum phosphates and serum alkaline phosphatase levels. We also tried to define the relationship between VD status and PTH levels in internationally adopted children.

## Methods

### Characteristics of the study population

We retrospectively gathered data provided by medical records of the Ethnopediatrics outpatient clinic of the “A. Gemelli” University Hospital in Rome, Italy. Inclusion criteria of our study were: being an internationally adopted child (age 1–15) and having one determination of 25(OH)D values during the first observation in our outpatient clinic from March 2007 to May 2016. The only exclusion criteria we applied was the lack of 25(OH)D measurement during the first observation. At the end of the assessment of the medical records, 859 children were included in the study.

Missing data about physical characteristics, due to the refusal of the child to undergo physical evaluation at the time of the first visit, were treated as “missing”, as well as missing data about clinical history of the child, due to a lacking anamnesis, were.

### Retrieving the data: Biochemical analysis methods and normal values

Blood samples were collected by venipuncture in the morning and routinely analyzed at the Hormone Laboratory (“A. Gemelli” University Hospital).

Serum 25(OH)D was measured by ChemiLuminescence ImmunoAssay (CLIA, Siemens) with an intra- and inter-assay variation of 5%. This method does not allow to differentiate D_2_ and D_3_ forms. The lower detection limit of the assay was 7 ng/mL. We entered in the database every value indicated as < 7 ng/mL within the test results as 6 ng/mL. Normal values are 31–100 ng/mL.

Serum intact parathyroid hormone (PTH) was assayed by ElettroChemiLuminescence ImmunoAssay (ECLIA, Roche) with an intra- and interassay CVs of 5% (normal values 9–65 pg/mL). Serum calcium (Ca) and phosphates (P) were determined by colorimetric assay (COBAS, Siemens). Serum calcium normal values are 8.8–10.8 mg/dl for all ages, while serum *P* values are 4–7 mg/dL when the child is younger than 10 years, or 2.5–4.5 mg/dL when the child is older than 10 years. Intra- and interassay CVs were 2.7 and 2.9%, respectively. Alkaline phosphatase (ALP) was measured by kinetic before 2011, normal values < 750 IU/L for children younger than 10 years, < 1000 IU/L for children older than 10 years. A kinetic photometric test IFCC (AMP) after 2011 (Siemens) was used instead after 2011, normal values were 40–300 IU/L for children aged < 13-years-old, while for children older than 13 years it is necessary to differentiate between males (normal values 40–309 IU/L) and females (normal values 40–187 IU/L). CVs intra- and interassay was 4% respectively.

Missing data, due to unavailability of reagents at certain time in our laboratory, are treated as “missing”.

### Assessment of 25-OH-D status

We chose to apply the Endocrine Society clinical practice guidelines cut-offs [28, 29]. Therefore, VD status was defined as severe deficiency when 25(OH)D was lower than 10 ng/ml, moderate deficiency when 25(OH)D value was between 10 and 20 ng/ml, mild deficiency when 25(OH)D value was between 20 and 30 ng/ml, or sufficiency when 25(OH)D was above 30 ng/ml.

Biochemical rickets was defined according to the following criteria: VDD (all categories) associated with increased ALP levels, normal or decreased calcium levels and normal or decreased phosphate levels.

### Statistical analyses

The population in study was divided into three climatic zones on the basis of their birth country: temperate zone (from circles to the latitude of 40°N/S), subtropical zone (from 40° N/S to the tropic of cancer/capricorn) and tropical zone (from Cancer’s tropic to Capricorn’s tropic).

Standing height was measured with a wall-mounted stadiometer, while standing body weight was measured with a mechanical scale. BMI was calculated using the Quetelet equation (Weight (kg)/Height (m)^2^). Height, weight, and BMI were expressed in centiles. BMI was categorized into age- and gender-specific percentiles for children and adolescents (WHO, 2006): i) BMI < 3° centile – severly underweight; ii) 3–10° centile – underweight; iii) BMI 11–85° centile – normal weight; iv) BMI 86–95° centile – overweight; v) BMI > 95° centile – obese.

Skin color was categorized into three groups: very fair/fair, intermediate, olive/brown. The Von Luschan’s chromatic scale was used to differentiate skin colors (0–13 on Von Luschan’s scale was categorized as “very fair/fair”; 14–20 as “intermediate”; 21–36 as “olive/brown”).

Four groups were arranged on the basis of the season of the blood sampling: winter (December 21 – March 20), spring (March 21 – June 20), summer (June 21 – September 20) and autumn (September 21 – December 20).

Descriptive and inferential statistics were carried out using the Statistical Package of Social Sciences (Chicago, IL, USA) for Windows software program version 22.0. Frequencies and percentages (%) were used to describe categorical variables (gender, macro-area, climatic zones, skin color, season at blood sampling, housing solution before adoption, category of BMI, VD status, PTH status, ALP status, Ca status, P status, biochemical rickets), while mean and standard deviation (mean ± SD) were used to express continuous variables (age on arrival in Italy, age at blood draw, time between the arrival and the blood draw, duration of the institutionalization, height, weight, BMI, 25(OH)D, PTH, ALP, Ca, P). We determined the normal distribution of continuous variables with a Kolmogorov-Smirnof test: the result permitted us to use parametric tests.

Differences among the categorical variables were tested for statistical significance using the Chi-square test. A *p* value < 0.05 was considered significant, Confidence Interval (CI) was 95%. Differences among mean values of continuous variables were tested for statistical significance using the Analysis of Variance (ANOVA) test and T test. A p value < 0.05 was considered significant, with a CI = 95%.

Post hoc analysis was performed for χ^2^ tests significant results, when a contingency table bigger than 2*2 was generated Bonferroni adjusted *p*-values were considered to determine which couple of categorical variables significantly differed.

A multivariate logistic regression was performed for exploring possible predictors of low 25(OH)D levels at a cut-off point of 30 ng/ml.

Variables to be entered in the multivariate model were chosen on the basis of the univariate analysis results (*p* <  0.25). To identify the best model a backward approach was developed, based on Likelihood Ratio test. The final model was assessed in terms of calibration and discrimination through Hosmer-Lemeshow and C-statistics.

ORs for proportions below these cut-offs and their confidence intervals are reported.

## Results

Characteristics of the population, season at blood drawing and mean values of the analytes investigated (25(OH)D, PTH, ALP, Ca, P) are resumed in Table 1. Detailed informations about post-hoc analyses are included in Additional file 1.

**Table 1:** Characteristics of the population included in the study

*Total*	859	100%
*Sex (n = 859)*		
Male	483	56.2%
Female	376	43.8%
*Macro-area of origin (n = 859)*		
Europe and Russian federation	256	29.8%
Latin America	231	26.9%
Asia and Indian subcontinent	223	26.0%
Africa	149	17.3%
*Climatic zone of origin (n = 859)*		
Tropical	438	51.0%
Sub-tropical	166	19.3%
Temperate	255	29.7%
*Housing solution before adoption (n = 828)*		
Foster home	70	8.5%
Foster family	65	7.9%
Institute	693	80.6%
*Mean duration of institutionalization (n = 750)*	3.00 years	SD ± 2.03 years
*Complexion (n = 859)*		
Very fair/Fair	290	33.8%
Intermediate	340	39.6%
Olive/Brown	229	26.7%
*Mean height (n = 793)*	109 cm	SD ± 21 cm
*Mean weight (n = 797)*	19.93 kg	SD ± 9.02 kg
*Mean BMI (n = 699)*	16.0	SD ± 2.28
*BMI status (n = 699)*		
Obese	59	8.4%
Overweight	67	9.6%
Normal	536	76.7%
Underweight	37	5.3%
*Mean age at the arrival in Italy (n = 808)*	5.31 years	SD ± 2.92 years
*Mean age at the blood draw (n = 859)*	5.61 years	SD ± 2.97 years
*Mean time from the arrival to the blood draw (n = 808)*	0.33 years	SD ± 0.44 years
*Season at blood draw (n = 859)*		
Spring	191	22.2%
Summer	149	17.3%
Fall	259	30.2%
Winter	260	30.3%
*Mean Vitamin D (n = 859)*	21.04 ng/mL	SD ± 11.15 ng/mL
*Vitamin D status (n = 859)*		
Severe Vitamin D Deficiency (< 10 ng/mL)	107	12.5%
Moderate Vitamin D Deficiency (10–20 ng/mL)	348	40.5%
Mild Vitamin D Deficiency (20–30 ng/mL)	276	32.1%
Normal Vitamin D (> 30 ng/mL)	128	14.9%
*Mean PTH (n = 822)*	32.24 pg/mL	SD ± 15.56 ng/mL
*PTH status (n = 822)*		
Increased (> 65 pg/mL)	24	2.8%
Normal (10–65 pg/mL)	795	96.7%
Decreased (< 10 pg/mL)	3	0.4%
*Mean ALP (n = 831)*	385.01 IU/L	SD ± 299.27 IU/L
*ALP status (n = 831)*		
Increased	201	24.2%
Normal	630	75.8%
*Mean Ca (n = 824)*	9.94 mg/dL	SD ± 0.45 mg/dL
*Ca status (n = 824)*		
Increased	18	2.2%
Normal	803	97.5%
Decreased	3	0.4%
*Mean P (n = 813)*	4.86	mg/dL
*P status (n = 813)*		
Increased	46	5.7%
Normal	731	89.9%
Decreased	36	4.4%

*BMI* body mass index, *PTH* Parathyroid hormone, *ALP* Alkaline phosphatase, *Ca* Serum calcium, *P* Serum phosphates

### Vitamin D mean value and status and characteristics of the population (Table 2)

We did not find any significant difference regarding differences between mean values in females and males (*p* = 0.594) regarding mean 25(OH)D values. On the other hand, we found a slightly significant influence of sex on Vitamin D status (*p* = 0.043). Post hoc analysis determined that sex was significantly related to VD status only when testing a severe versus a moderate VDD, with female children having an increased risk of developing severe VDD than male children (OR 0.55, 95% CI 0.36–0.86).

**Table 2:** Vitamin D status and characteristics of the population

		Vitamin D Status				
		*Severe VDD (< 10 ng/mL)*	*Moderate VDD (10–20 ng/mL)*	*Mild VDD (20–30 ng/mL)*	*Normal VD (> 30 ng/mL)*	*p values*
Sex *(n = 859)*	Male	49 (10.1%)	211 (43.7%)	149 (30.9%)	74 (15.3%)	0.043
	Female	58 (15.4%)	137 (36.4%)	127 (33.8%)	54 (14.4%)	
BMI category *(n = 699)*	Underweight	2 (5.4%)	17 (45.9%)	14 (37.8%)	4 (10.8%)	0.521
	Normal weight	78 (14.5%)	235 (43.8%)	160 (29.9%)	63 (11.8%)	
	Overweight	11 (16.4%)	21 (31.3%)	26 (38.8%)	9 (13.4%)	
	Obese	7 (11.9%)	29 (49.1%)	18 (30.5%)	5 (8.5%)	
Skin color *(n = 859)*	Very fair/fair	36 (12.4%)	115 (39.7%)	98 (33.8%)	41 (14.1%)	< 0.001
	Intermediate	26 (7.6%)	128 (37.7%)	121 (35.6%)	65 (19.1%)	
	Olive/Brown	45 (19.6%)	105 (45.9%)	57 (24.9%)	22 (9.6%)	

χ^2^-test was used to determine statistically significant relationships among categorical variables

Bonferroni-adjusted P value was uses to determine if there were statistically significant differences among groups

*VDD* Vitamin D Deficiency; *VD* Vitamin D; *BMI* Body Mass Index

We did not find any statistically significant difference between 25(OH)D mean values and BMI categories (*p* = 0.477). The difference is not statistically significant either when applied to Vitamin D status (*p* = 0.521).

A statistically significant difference was found for skin color (*p* <  0.001) regarding mean 25(OH)D values. Mean 25(OH)D value was 18.00 ng/mL (SD ± 10.78 ng/mL) for children included in the group “olive to brown”; 20.86 ng/mL (SD ± 10.12 ng/mL) for children included in the group “very fair to fair”; 23.24 ng/mL (SD ± 11.76 ng/mL) for children included in the group “intermediate”. This difference remains statistically significant when considering Vitamin D Status (p <  0.001). However, post hoc analysis highlighted that statistically significant differences only exist with regards to intermediate skin color when compared to olive/brown skin color. As a matter of fact, the character “olive/brown” skin resulted to be predictive of a worse VD status both when the severe VDD status is compared to mild VDD status (OR 0.27, 95% CI 0.151–0.489) and when the severe VDD status is compared to normal VD status (OR 0.196, 95% CI 0.101–0.391).

More detailed informations about the results above illustrated can be found in Table 2 and in Additional file 1.

Time from the arrival to the first evaluation was not found to be statistically related with 25(OH)D mean values (*p* = 0.388) and Vitamin D Status (*p* = 0.912). Age at the arrival in Italy was significantly associated both with 25(OH)D mean values (*p* <  0.001) and Vitamin D status (p <  0.001). Age at the blood draw association with 25(OH)D mean values (*p* = 0.049) was marginally significant, but it had a highly statistically significative relationship with Vitamin D status (*p* <  0.001).

Table 3 shows mean time from the arrival to first evaluation, age at the arrival and age at first evaluation in relation to Vitamin D Status. Table 4 shows age at the arrival in Italy in years (mean ± SD) per Vitamin D Status, and its relations with sex, macro-area, season at blood draw, housing solution and BMI status.

**Table 3:** Vitamin D status and mean time from the arrival to 1st evaluation, age at the arrival and age at 1st evaluation

	Vitamin D Status				
	*Severe VDD (< 10 ng/mL)*	*Moderate VDD (10–20 ng/mL)*	*Mild VDD (20–30 ng/mL)*	*Normal VD (> 30 ng/mL)*	*p values*
Time from the arrival in Italy to 1st evaluation *(n = 808) mean ± SD (years)*	0.32 ± 0.47	0.34 ± 0.41	0.32 ± 0.47	0.32 ± 0.47	0.912
Age at the arrival in Italy *(n = 808) mean ± SD (years)*	6.31 ± 2.84	5.71 ± 2.77	5.09 ± 2.79	3.86 ± 3.07	< 0.001
Age at 1st evaluation *(n = 859) mean ± SD (years)*	6.69 ± 2.85	6.03 ± 2.79	5.36 ± 2.77	4.11 ± 3.15	< 0.001

ANOVA test was used to determine statistical significance

**Table 4:** Age at the arrival in Italy in years (Mean ± standard deviation) per Vitamin D status

		Vitamin D status			
		*Severe VDD*	*Moderate VDD*	*Mild VDD*	*Normal*
Sex	*Male*	6.15 ± 2.77	5.50 ± 2.75	4.91 ± 2.59	3.74 ± 2.75
	*Female*	6.44 ± 2.92	6.06 ± 2.78	5.32 ± 3.01	4.02 ± 3.49
Macro-area	*Africa*	5.18 ± 2.48	4.85 ± 2.19	4.24 ± 2.51	3.16 ± 2.64
	*Europe*	6.16 ± 2.84	5.80 ± 2.71	5.28 ± 2.43	4.88 ± 3.40
	*Asia*	6.32 ± 2.99	4.14 ± 2.15	3.25 ± 2.39	1.82 ± 1.29
	*Latin America*	8.08 ± 2.37	7.16 ± 2.80	6.99 ± 2.37	6.31 ± 2.68
Season at blood draw	*Spring*	6.46 ± 2.83	6.09 ± 2.85	3.87 ± 2.49	2.44 ± 1.95
	*Summer*	5.77 ± 3.22	5.24 ± 2.61	4.62 ± 2.59	4.55 ± 3.73
	*Fall*	5.70 ± 2.96	5.57 ± 2.61	5.59 ± 2.72	3.85 ± 2.59
	*Winter*	6.46 ± 2.85	5.70 ± 2.90	5.59 ± 2.99	2.68 ± 3.86
Housing solution	*Foster home*	7.37 ± 3.55	6.73 ± 2.86	6.62 ± 2.67	4.64 ± 2.78
	*Foster family*	8.86 ± 2.96	6.81 ± 3.18	5.40 ± 2.43	6.79 ± 4.26
	*Orphanage*	6.09 ± 2.85	5.73 ± 2.79	4.88 ± 2.78	3.57 ± 3.07
BMI Status	*Underweight*	2.87 ± 0.49	5.07 ± 2.00	5.17 ± 2.03	5.76 ± 5.99
	*Normal weight*	6.44 ± 2.56	6.08 ± 2.63	5.66 ± 2.39	5.42 ± 2.76
	*Overweight*	7.75 ± 3.01	6.65 ± 2.64	5.99 ± 2.41	4.18 ± 2.07
	*Obese*	6.71 ± 3.44	6.28 ± 2.26	7.19 ± 2.81	6.56 ± 2.12

*VDD* Vitamin D Deficiency, *BMI* Body Mass Index

**Fig. 1:**
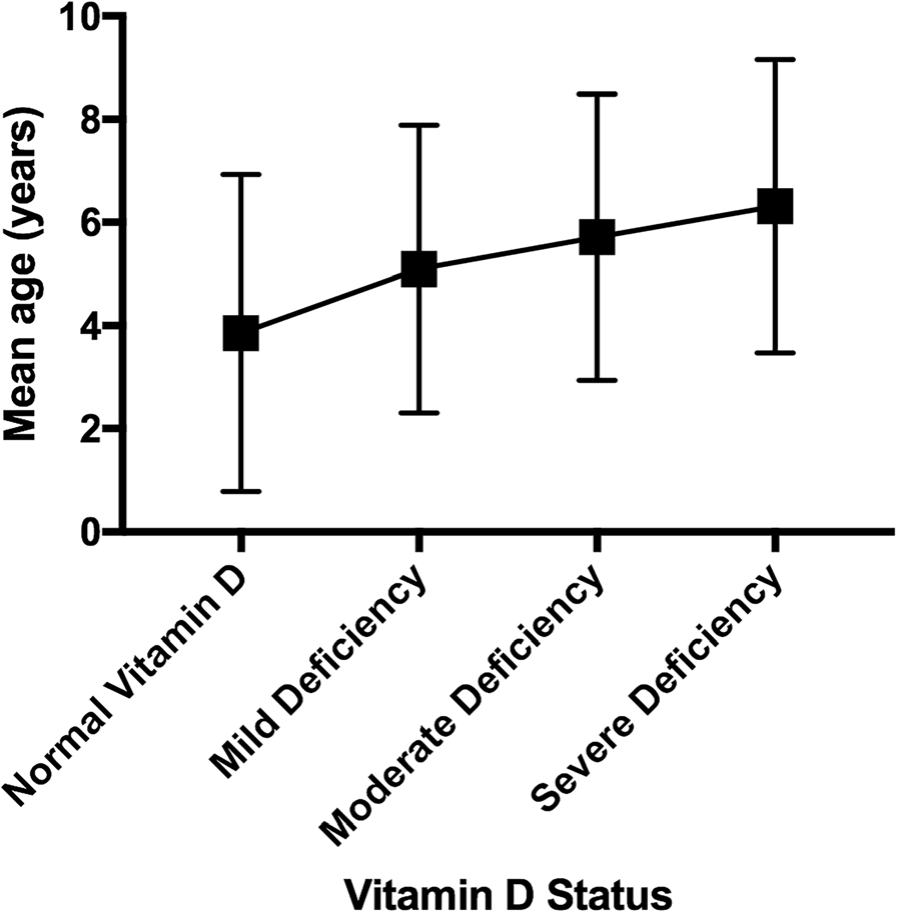
Vitamin D status in relation to internationally adopted children mean age. Vitamin D status seems to be inversely related to internationally adopted children mean age. As a matter of fact, in our cohort a worst status can be observed in older children

**Fig. 2:**
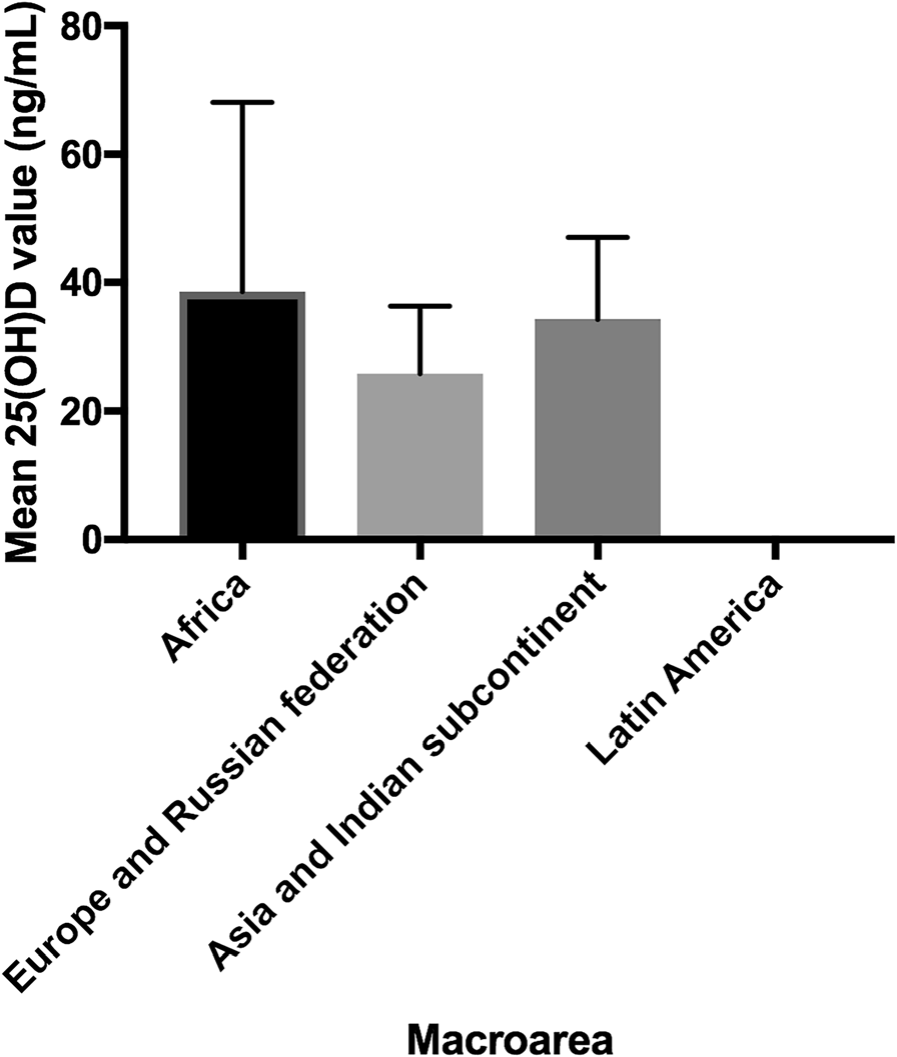
Mean 25(OH)Vitamin D values in children younger than 1 year of age per macroarea of origin. We observed higher Vitamin D values in children < 1 yo who came from Africa and Asia, compared with those coming from Europe. In our cohort, no children were internationally adopted from Latin America when younger than 1 year of age

Figure 1 shows mean age at the arrival, in years, and its relationship with Vitamin D Status, while fig. 2 shows mean 25(OH)D values for children aged < 1 year and their relationship with macro-area of origin.

### Vitamin D mean value and status, geographical origin and social environment **(**Table 5**)**

Macro-area of origin was found to have a statistically significant relationship with 25(OH)D mean values (*p* = 0.001) and Vitamin D status (p <  0.001). Mean 25(OH)D value in children coming from Africa was 19.04 ng/mL (SD ± 11.67 ng/mL); in children coming from Latin America was 20.18 (SD ± 7.56 ng/mL); in children coming from Europe and Russian Federation was 21.00 ng/mL (SD ± 10.27 ng/mL); in children coming from Asia and Indian subcontinent was 23.31 ng/mL (SD ± 14.17 ng/mL).

**Table 5:** Vitamin D status, geographical origin and social environment

		Vitamin D Status				
		*Severe VDD (< 10 ng/mL)*	*Moderate VDD (10–20 ng/mL)*	*Mild VDD (20–30 ng/mL)*	*Normal VD (> 30 ng/mL)*	*p values*
Macro-area *(n = 859)*	*Europe*	24 (16.1%)	68 (45.6%)	39 (26.2%)	18 (12.1%)	< 0.001
	*Latin America*	31 (12.1%)	103 (40.2%)	85 (33.2%)	37 (14.5%)	
	*Asia*	34 (14.2%)	67 (30.1%)	73 (32.7%)	49 (22.0%)	
	*Africa*	18 (7.8%)	110 (47.6%)	79 (34.2%)	24 (10.4%)	
Climatic zone *(n = 859)*	*Temperate*	31 (12.2%)	102 (40.0%)	85 (33.3%)	37 (14.5%)	0.002
	*Subtropical*	34 (20.5%)	68 (41.0%)	52 (31.3%)	12 (7.2%)	
	*Tropical*	42 (9.7%)	178 (40.6%)	139 (31.7%)	79 (18.0%)	
Housing solution *(n = 828)*	*Foster home*	8 (11.4%)	24 (34.4%)	19 (27.1%)	19 (27.1%)	0.008
	*Foster family*	6 (9.2%)	37 (56.9%)	18 (27.7%)	4 (6.2%)	
	*Orphanage*	90 (13.0%)	275 (39.7%)	227 (32.8%)	101 (14.5%)	
Season of first blood draw *(n = 859)*	*Spring*	29 (15.2%)	94 (49.2%)	50 (26.2%)	18 (9.4%)	< 0.001
	*Summer*	3 (2.8%)	41 (38.3%)	56 (52.3%)	49 (45.8%)	
	*Fall*	18 (6.9%)	101 (39.0%)	103 (39.8%)	37 (14.3%)	
	*Winter*	57 (21.9%)	112 (43.1%)	67 (25.8%)	24 (9.2%)	

χ^2^-test was used to determine statistically significant relationships among categorical variables

Post hoc analysis highlighted that statistically significant differences existed within children coming from Asia and children coming from Africa. As a matter of fact, children coming from Africa tent to have more “intermediate” values than children coming from Asia. The result was statistically significant when comparing severe VDD status with moderate VDD status and moderate VDD status with normal VD status, with different outcomes. Children coming from Africa tend to have higher values of VD when comparing the severe and the moderate VDD status (OR 3.101, 95% CI 1.659–5.927), but lower VD values when comparing the moderate VDD status with the normal VD status (OR 0.298, 95% CI 0.171–0.531).

Details about the results above described can be found in Table 5 and Additional file 1.

Climatic-zones of origin were also found to have a significant influence over 25(OH)D mean values (*p* <  0.001) and Vitamin D Status (*p* = 0.002). Mean 25(OH)D value in children coming from a tropical area was 22.27 ng/mL (SD ± 12.24 ng/mL), in children coming from a subtropical area was 17.83 ng/mL (SD ± 8.51 ng/mL), while in children coming from a temperate zone was 21.00 ng/mL (SD ± 10.28 ng/mL).

Post hoc analyses highlighted that statistically significant differences existed only for those children coming from subtropical areas when compared to children coming from tropical areas. As a matter of fact, children coming from a tropical area tend to have higher VD values than children coming from a subtropical area when comparing the severe VDD status with normal VD status (OR 5.329, 95% CI 2.512–11.41).

Detailed informations about the results above described can be found in Table 5 and Additional file 1.

Even housing solution had a significant relationship with 25(OH)D mean values (*p* = 0.016) and vitamin D status (*p* = 0.008). Mean 25(OH)D values in children who were guest of a foster house were 24.21 ng/ml (SD ± 16.13 ng/mL), in children housed in a foster family were 18.79 ng/mL (SD ± 6.72 ng/mL), while in children who were guest of an orphanage were 20.95 ng/mL (SD ± 10.92 ng/mL).

Post hoc analyses highlighted how actually the only significant difference can be found comparing children coming from foster homes with children coming from foster families. Children coming from foster families seem to be more at risk of a worst VD status, especially when comparing moderate VDD status with normal VD status (OR 0.137, 95% CI 0.049–0.453).

Detailed data about the results above described can be found in Table 5 and Additional file 1.

### Vitamin D mean values and status and their relations with season of blood sampling

Season at blood sampling was significantly related both with 25(OH)D mean values (*p* <  0.001) and Vitamin D status (p <  0.001). Mean 25(OH)D value in children having their blood drawn in spring was 18.85 ng/mL (SD ± 10.39 ng/mL), in children undergoing blood draw in summer was 26.82 ng/mL (SD ± 11.08 ng/mL), in children who were firstly evaluated during fall was 22.28 ng/mL (SD ± 10.10 ng/mL) and in children having their first examination during winter was 18.10 ng/mL (SD ± 11.32 ng/mL).

Post hoc analyses highlighted how during summer the risk of VDD is substanstially decreased, compared to all the other seasons. Interestingly, a statistically significant difference can be found between children observed during spring and children observed during fall, when severe VDD status is compared to mild VDD status. Children observed during fall tend to have a better VD status than children observed during spring (OR 3.319, 95% CI 1.656–6.715).

Table 5 shows Vitamin D status in relation to macroarea, climatic zone of origin, housing solution and season at blood sampling. Detailed data about post hoc analyses can be found in Additional file 1.

### Vitamin D mean values and status and their relations with other factors of the bone metabolism

PTH status was not significantly related to 25(OH)D mean values (*p* = 0.609) and Vitamin D Status (*p* = 0.086).

ALP status was not significantly associated with 25(OH)D mean values (*p* = 0.593), but it was significantly associated with Vitamin D status (*p* = 0.001). Mean 25(OH)D value in children having a normal ALP was 20.99 ng/mL (SD ± 10.87 ng/mL), while in children having an increased ALP it was 21.48 ng/mL (SD ± 12.30 ng/mL).

Post hoc analysis revealed that this statistically significant difference is only real when comparing severe VDD status with mild VDD status, with children having an increased ALP being at more risk of having a worse VD status (OR 0.4562, 95% CI 0.2737–0.7773).

Detailed data about post hoc analyses can be found in Additional file 1.

Ca status was not significantly related to 25(OH)D mean values (*p* = 0.338) or Vitamin D status (*p* = 0.296).

P mean values were significantly associated to 25(OH)D mean values (*p* = 0.004) and to Vitamin D status (*p* = 0.012). P status was equally significantly related to 25(OH)D mean values (*p* = 0.008) and to Vitamin D status (*p* = 0.014). 25(OH)D mean value in children having a normal P was 21.22 ng/mL (SD ± 11.14 ng/mL), in children having a decreased P was 23.06 ng/mL (SD ± 12.61 ng/mL).

Table 6 shows Vitamin D status in relation to PTH status, ALP status, Ca status and P status.

**Table 6:** Vitamin D status and its relations with PTH status, ALP status, Ca status and P status

		Vitamin D Status				
		*Severe VDD (< 10 ng/mL)*	*Moderate VDD (10–20 ng/mL)*	*Mild VDD (20–30 ng/mL)*	*Normal VD (> 30 ng/mL)*	*p values*
PTH status *(n = 822)*	*Normal* (< 10 pg/mL)	94 (11.8%)	323 (40.6%)	262 (33.0%)	116 (14.6%)	0.086
	*Decreased* (10–65 pg/mL)	0 (0.0%)	0 (0.0%)	3 (100%)	0 (0.0%)	
	*Increased* (> 65 pg/mL)	3 (12.5%)	14 (58.3%)	3 (12.5%)	4 (16.7%)	
ALP status *(n = 831)*	*Normal* ^a^	69 (10.9%)	259 (41.1%)	218 (34.7%)	84 (13.3%)	0.001
	*Increased* ^a^	34 (16.9%)	76 (37.8%)	49 (24.4%)	42 (20.9%)	
Ca status *(n = 824)*	*Normal* (8.8–10.8 mg/dL)	100 (12.5%)	324 (40.3%)	261 (32.5%)	84 (13.3%)	0.296
	*Decreased* (< 8.8 mg/dL)	0 (0.0%)	3 (100%)	0 (0.0%)	0 (0.0%)	
	*Increased* (> 10.8 mg/dL)	1 (5.6%)	5 (27.8%)	8 (44.4%)	4 (22.2%)	
P status *(n = 813)*	*Normal* ^b^	84 (11.5%)	293 (40.1%)	249 (34.1%)	105 (14.3%)	0.014
	*Decreased* ^b^	4 (11.1%)	16 (44.5%)	7 (19.4%)	9 (25.0%)	
	*Increased* ^b^	12 (26.1%)	20 (43.5%)	11 (23.9%)	3 (6.5%)	

χ^2^-test was used to determine statistically significant relationships among categorical variables

^a^ Normal values, depending on the date of the blood draw and the age/sex of the patient, are accurately described within the section “Subjects and Methods”;

^b^ Normal values, depending on the age of the patient, are accurately described within the section “Subjects and Methods”

### Biochemical rickets

Only 811 children (94.4%) out of 859 had the complete blood tests panel for the definition of biochemical rickets. The analysis performed permitted to identify 129 children affected by biochemical rickets as defined in the “*Subjects and Methods*” section. 28 children (21.7%) had severe VDD and an increased ALP, associated with normal or reduced Ca and P. 61 children (47.3%) had moderate VDD and an increased ALP, associated with normal or reduced Ca and P. 40 children () had mild VDD and an increased ALP, associated with normal or reduced Ca and P.

Table 7 resumes details about children affected by biochemical rickets.

**Table 7:** Biochemical rickets: number of children affected and 25(OH)D mean values

			P status		
			*Normal* ^*b*^ *(25-OH-D mean value ± SD)*	*Decreased* ^*b*^ *(25-OH-D mean value ± SD)*	*Total*
Vitamin D Status	*Severe deficiency + increased ALP*	*Normal Ca* ^c^	26 (7.20 ± 1.46 ng/mL)	2 (6.65 ± 0.92 ng/mL)	129
		*Decreased Ca* ^c^	0	0	
	*Moderate deficiency + increased ALP*	*Normal Ca* ^c^	59 (15.30 ± 2.93 ng/mL)	1 (15.00 ± NA ng/mL)	
		*Decreased Ca* ^c^	1 (17.70 ± NA ng/mL)	0	
	*Mild deficiency + increased ALP*	*Normal Ca* ^c^	40 (24.96 ± 2.89 ng/mL)	0	
		*Decreased Ca* ^c^	0	0	

*ALP* Alkaline phosphatase; *Ca* Serum calcium; *P* Serum phosphate; *25-OH-D* 25-hydroxylate-Vitamin D

^a^ Normal values, depending on the date of the blood draw and the age/sex of the patient, are accurately described within the section “Subjects and Methods”;

^b^ Normal values, depending on the age of the patient, are accurately described within the section “Subjects and Methods”

^c^ Normal values can be found in Table 3 and within the section “Subjects and Methods”

### Multivariate analysis

We entered the statistically significant variables (*p* <  0.25) in relation to Vitamin D status (sex, skin color, macroarea of origin, climatic zone of origin, season at first evaluation, housing solutions, ALP status, P status, Age at arrival in Italy, Age at first visit) in a multiple logistic regression model. At the end of the assessment, skin color (*p* = 0.011), season at first blood draw (*p* <  0.001), housing solution (p <  0.001), ALP status (*p* = 0.025) and the age at the first blood draw (p <  0.001) had a statistically significant association with Vitamin D Status.

**Fig. 3:**
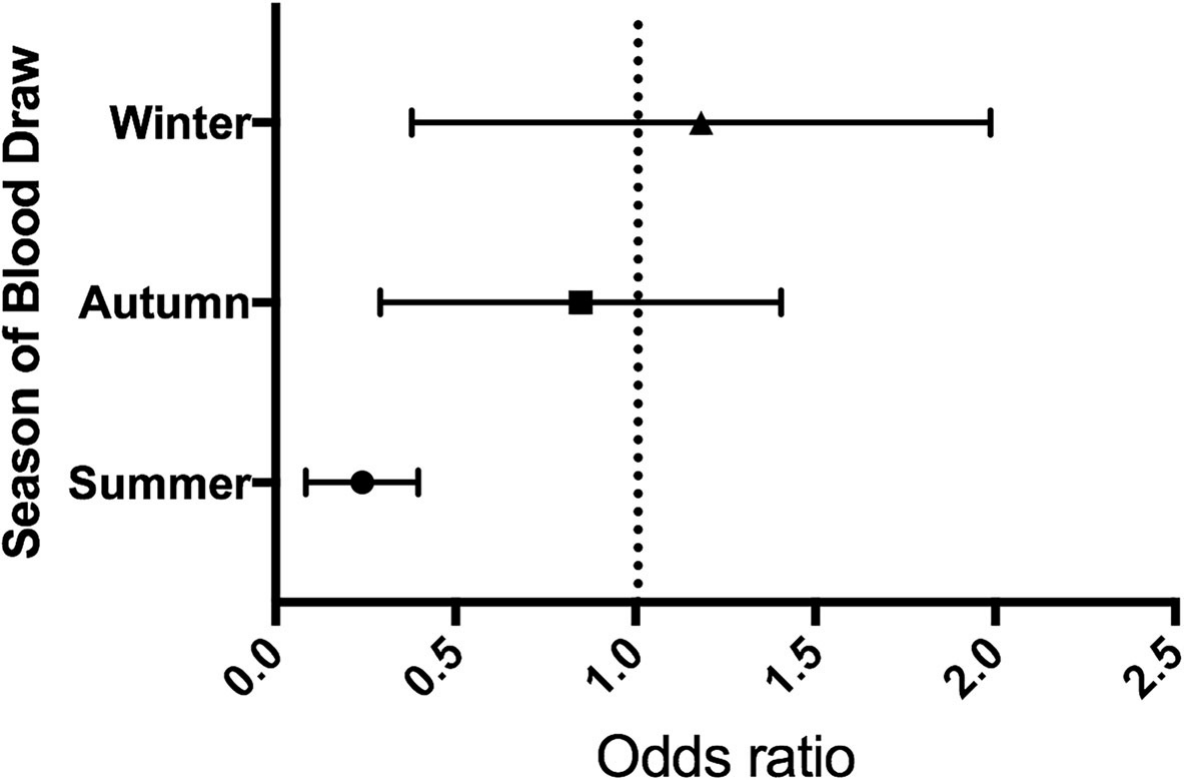
Risk of finding a vitamin D (VDD) deficiency according to season of blood draw. When using spring as a baseline, the only significative association is a lower risk of finding a VDD when the blood draw is performed during summer. Autumn is associated with a non-significant slightly lower risk, while Winter with a non-significant slightly higher risk

Table 8 shows details about the multiple logistic regression analysis final results. Figure 3 shows the risk of developing hypovitaminosis D during the different seasons when using spring as baseline.

**Table 8:** Multiple logistic regression results: showing predictors of developing Vitamin D Deficiency through Odds Ratios (ORs), 95% Confidence Intervals (CIs) and statistical significance of variables (*p* value)

Variable	Value	OR	95% CI	*p* value
Skin color	*Very fair / fair (ref)*			
	*Intermediate*	1.014	0.590–1.743	0.96
	*Olive / Brown*	2.387	1.261–4.519	0.08
Season at 1st evaluation	*Spring (ref)*			
	*Summer*	0.207	0.104–0.410	< 0.001
	*Fall*	0.726	0.362–1.455	0.366
	*Winter*	1.000	0.485–2.063	1.000
Housing solution	*Foster home (ref)*			
	*Foster family*	0.252	0.125–0.507	< 0.001
	*Orphanage*	3.971	1.971–8.001	< 0.001
ALP status	*Normal* ^*a*^ *(ref)*			
	*Increased* ^*a*^	0.560	0.338–0.930	0.025
Age at 1^st^ evaluation	*Continuous variable*	1.238	1.140–1.345	< 0.001

*ALP* Alkaline phosphatase

^a^ Normal values are extensively described in the “Subjects and methods” section

## Discussion

Migrant children are a population of great interest nowadays, and their health problems—infectious ones, but also not-infectious ones—are a source of interesting discussions for pediatricians all over the world. Internationally adopted children are a controlled sub-population of the migrant one, thus it could offer an insight on a larger population – though with a lot of differences regarding travelling, housing solutions and dietary habits and deprivations.

Vitamin D is an important pro-hormone, classically associated with bone health, but recently found to be involved in immune system and neuro-psychological disorders, and cardiovascular risk. It can be seen how a D hypovitaminosis could put at risk the entire well-being of a child [1–5].

Our study, carried on for nine years, tried to test some of the well-known predictors for the Vitamin D deficiency in the internationally adopted children population and the relationships existing between vitamin D and other factors implied in the bone health, such as parathyroid hormone, alkaline phosphatase, free calcium and phosphates.

From January 2007 to December 2015, 29,866 children arrived in Italy through an international adoption, from Eastern Europe countries above all, followed by Latin America, Asia and Africa [30]. The cited report offers only details about 2014 and 2015, but it specifies that the percentages reported are similar to those previously observed. During this period, adopted male children were more than the female ones (58.3% vs 41.7%; ratio = 1.4:1), with a mean age at the arrival of 5.9 years (SD not specified).

The population we observed had a similar composition to that of the entire “internationally adopted children” Italian population: male to female ratio was 1.3:1, with a prevalence of children coming from Eastern Europe countries and a mean age at the arrival of 5.31 years (SD ± 2.92 years).

We did not find a statistical difference of mean 25(OH)D values between sexes. However, a slight statistical significance was found with regards to the Vitamin D status, with a slight higher percentage of females affected by VDD than males. Female children are, as a matter of fact, more interested by severe VDD and mild VDD than male children, while male children seem to be more affected by moderate VDD than females (Table 2 and Additional file 1).

A reason for this evidence could be a higher BMI in female children than males, a justification often found by other studies, highlighting how a higher BMI is directly related to the risk of developing VDD, as VD is a lipophilic vitamin and a higher fat mass increase its distribution volume [31–35].

Our results, though, are opposite to those showed by other studies in which VDD was found to be more prevalent among males than in females, or it showed no significant relationship with sex at all [11, 36–39].

In our population, prevalence of VDD is significantly related to the age at the arrival in Italy and to the age at first evaluation. Figure 1 shows the mean age per group of Vitamin D status, highlighting that in our population a higher mean age is related to a worst Vitamin D status.

The negative association of age and serum 25(OH)D in children has already been described in literature. According with Mansbach et al., this might be because of the difference in duration of sun exposure, while Gustafson et al. sustain that it is caused by the rapid growth spurt, or “catch-up growth” the children reach out in the first year after adoption [7, 40].

Catch-up growth is a process similar to the rapid growth experienced by infants and toddlers, when use of VD supplement is customary for the prevention of rickets [41]. Therefore, during this period internationally adopted need larger amounts of dietary or supplemental iron and VD than those a child of the same age would normally need.

Interestingly, in our population high 25(OH)D concentrations were observed in children aged < 1 year regardless of the area of origin (Fig. 1). This is most likely attributable to the use of fortified formula milk during the period spent in an institution and by pediatricians’ increasing awareness of the hypovitaminosis D issue once they arrive in Italy. As reported by other studies, this relation might lead to nullify the expected effects of an adoptee’s birth country on VD level [35, 40].

Our study showed a strong association between skin color and Vitamin D Status, that only remained significant for the value “olive-brown skin” after the multivariate analysis (logistic regression). Our data showed that children with olive to brown skin had a statistically significant 2-fold higher risk of having hypovitaminosis D than children with fair skin (OR 2.387, Table 8). These results agree with those reported by an Italian study by Franchi et al. evaluating 1.374 children living in the north of Italy. The study observed that ethnicity was a strong predictor of 25(OH)D levels, reporting a high prevalence of hypovitaminosis D in African, North African, and Indian children living in Italy (74.8, 81.2, 89.7, and 76.0%, respectively) [25]. Other studies reported the same results. A severe 25(OH)D deficiency associated to a proximal myopathy was observed in recent immigrants from Palestine, Pakistan and India to Northern Europe develop due to the limited effect of sunshine and a low dietary VD intake [37]. Hintzpeter [35] showed that Turkish and Arab-Islamic participants of both sexes as well as Asian and African girls were found to be at high risk for VDD, with the highest OR for African girls (7.8) [35]. Similarly, data from NHANES III showed that adolescents of African origin had an increased risk of VDD (OR 8.6) compared with white adolescents [15].

These consistent observations can lead us to conclude that a spontaneous recovery of VD status is unlikely in an adopted child arrived in Italy with VDD, independently from their ethnicity.

In our study *macro area* of origin was significantly associated with VD status (*p* < 0.001). Basing our observation on percentages alone, VDD seems to have been more common in Africa than in Latin America or Europe and Russian Federation. If statistically significant, our results would differ from those showed by Chiappini et al., whose study highlighted the highest prevalence of VDD in children coming from the Russian federation [26]. However, post hoc analyses highlighted that the real statistically significant differences are only between children coming from Asia and children coming from Africa. See Additional file 1 and the “results” section for a more detailed description of this difference. Thus, it is not possible to conclude that VDD is more common in children coming from Africa than in children coming from Latin America or Europe and Russian Federation.

As previously said with regards to skin color, it is thought that occasional exposure to sunlight provides most of the VD requirement of the human population [42]. However, skin synthesis of VD may not compensate for the low nutritional intake in Europe, because of the relationship occurring between its latitude and the rake angle of solar radiations. As a matter of fact, Webb et al. [43] demonstrated that the photosynthesis of pre-VD is nearly impossible during winter months at latitudes similar to those encasing Europe. A high prevalence of hypovitaminosis D combined with high PTH levels and lower bone mineral density has recently been reported in Finnish girls and Italian children and adolescents, suggesting that Italian children are at risk for this condition, as well as other European populations [44–46]. Moreover, another Italian study by Vierucci et al., showed that no VD is produced in central Italy during late fall, winter and the beginning of spring because of sun exposure [38, 39].

The prevalence of VDD, even at southern latitudes, was remarkable, supporting other reports suggesting that living in sunny areas of the world does not necessarily protect children from VDD [39, 43, 47–50].

Our work highlighted that the risk of developing hypovitaminosis D was significantly higher in children coming from foster families and orphanages, compared to those coming from foster families. This relationship was still statistically significant after multivariate analysis.

It is a well-demonstrated knownledge that children in foster care experience high rates of child abuse, emotional deprivation, and physical neglect than children living with their families [51–53]. Moreover, it is plausible to think that children in orphanages likely do not spend much time outdoors and that they lack adequate sun exposure. Another reason for the association with VDD could be that as children grow up in institutional care, they shift from a diet of vitamin D–fortified formula milk to cooked food, which may not be fortified with vitamin D.

According to other studies, we observed how the season of blood sampling could be considered a predictor of the Vitamin D Status [26, 54]. As a matter of fact, it is only natural to find out that a pro-hormone which synthesis is related to sun-exposure has higher levels during summer than during winter. It has been shown that even minimal exposure of skin to sunlight, is adequate to reach VD sufficiency in US and Central Europe, and a number of studies demonstrated that both serum concentrations of 25(OH)D and other biomarkers of bone health status increase during summer only to reduce during winter, while PTH behavior is the exact opposite [55–63].

Our data highlighted how drawing a blood sample during summer was a protective factor against hypovitaminosis D, when compared to spring (Fig. 1). OR for summer shows a reduction of the risk of approximately the 80%.

In our study, almost half the children had normal Ca, P, ALP and PTH levels. This data differs from other pediatric studies, which show a percentage way lower [23, 64]. Normal serum calcium works as an inhibitory signal for parathyroids, stopping PTH secretion, as described by Steingrimsdottir and Aggarwal [65, 66].

Our study differs from other studies on the same matter because of the interest taken in showing the relationship existing between vitamin D and parathyroid hormone. Only a very small percentage of our population was affected by hyperparathyroidism, but a significant data was that we could observe elevated PTH levels in children having 25(OH)D levels over 30 ng/mL and normal calcium, a finding deserving further studies: was it a primary hypersecretion or a signal of an altered bone metabolism?

We also tried to identify the PTH inflection point for this population. With this term, other studies indicated the mean 25(OH)D value at which PTH begins to increase. We did not succeed, probably because of the small number of children with an increased PTH included in our population and the extreme variability of values at which PTH was found to increase.

Biochemical rickets affected a significant percentage of our study population. Surprisingly, even children with only mild VDD were affected by it (Table 8). It is though that rickets is confined to children exposed to extreme undernourishment but, as a matter of fact, it can be also observed in western countries, because of the growing epidemia of childhood obesity and the previously discussed relationship of high body mass index and fat mass with VDD [67].

### Limits – Strenghts

Our study was limited by the lack of data on dietary intake of calcium and vitamin D. Type of nutrition in the first few months of life can deeply influence values of VD. Some of these infants and toddlers lived with their biological families prior to adoption and might have been breast-fed (low VD intake, limited Ca absorption); however, many international adoptees spent most of their life in orphanages or with foster families and were primarily formula fed (high VD intake, maximized Ca absorption).

Additionally, we could not retrieve any information about sunshine exposure, use of sun protections, medical conditions (i.e renal and/or hepatic failure), use of hypovitaminizing drugs, use of concealing clothing.

However, our study also has some strengths: it shows the irrelevance of PTH as a predictor of VDD, and identifies, through a multivariate analysis, some of the factors a pediatrician cannot miss when evaluating an internationally adopted child for the first time.

## Conclusions

The study indicates a widespread global VD insufficiency compared with proposed threshold levels. This high prevalence implies that higher doses than currently recommended would be needed to reach country specific “desirable” levels.

Our results reinforce the importance of an early evaluation of serum 25(OH)D levels in internationally adopted children to start the adequate strategy of VD supplementation/treatment, avoiding further deterioration of VD status, particularly in older, dark skin children arrived in Italy during winter and spring period.

## Additional file

Additional file 1: Post hoc analyses. (DOCX 17 kb)

## Abbreviations

25(OH)D: 25-hydroxy-vitamin D
ALP: Alkaline phosphatase
Ca: Calcium
P: Phosphorus
PTH: Parathormone
VD: Vitamin D
VDD: Vitamin D deficiency

## References

[1] Buonsenso D, Focarelli B, Scalzone M, Chiaretti A, Gioè C, Ceccarelli M, Valentini P (2012). Chest wall TB and low 25-hidroxy-vitamin D levels in a 15-month-old girl. It J Pediatr.

[2] Valentini P, Gargiullo L, Ceccarelli M, Ranno O (2012). Health status of internationally adopted children. The experience of an Italian “GLNBI” paediatric Centre. It J Publ health.

[3] Di Rosa M, Malaguarnera G, De Gregorio C, Palumbo M, Nunnari G, Malaguarnera L (2012). Immuno-modulatory effects of vitamin D3 in human monocyte and macrophages. Cell Immunol.

[4] Pinzone MR, Nunnari G (2015). Prevalence of comorbidities in a cohort of women living with HIV. Infect Dis Trop Med.

[5] Bruno R, Scuderi D, Locatelli ME, Pampaloni A (2017). Prevalence of micronutrients deficiencies in a cohort of HIV-positive individuals on ART. Infect Dis Trop Med.

[6] Holick MF, Chen TC (2008). Vitamin D deficiency: a worldwide problem with health consequences. Am J Clin Nutr.

[7] Mansbach JM, Ginde AA, Camargo CAJ (2009). Serum 25-hydroxyvitamin D levels among US children aged 1 to 11 years: do children need more vitamin D?. Pediatrics.

[8] Greer FR (2008). 25-Hydroxyvitamin D: functional outcomes in infants and young children. Am J Clin Nutr.

[9] Misra M, Pacaud D, Petryk A, Collett-Solberg PF, Kappy M (2008). Vitamin D deficiency in children and its management: review of current knowledge and recommendations. Pediatrics.

[10] Ross AC, Manson JE, Abrams SA, Aloia JF, Brannon PM, Clinton SK, Durazo-Arvizu RA, Gallagher JC, Gallo RL, Jones G, Kovacs CS, Mayne ST, Rosen CJ, Shapses SA (2011). The 2011 report on dietary reference intakes for calcium and vitamin D from the Institute of Medicine: what clinicians need to know. J Clin Endocrinol Metab.

[11] Thompson RM, Dean DM, Goldberg S, Kwasny MJ, Langman CB, Janicki JA (2017). Vitamin D insufficiency and fracture risk in urban children. J Pediatr Orthop.

[12] Hutchison FN, Bell NH (1992). Osteomalacia and rickets. Semin Nephrol.

[13] Gordon CM, Feldman HA, Sinclair L, Williams AL, Kleinman PK, Perez-Rossello J, Cox JE (2008). Prevalence of vitamin D deficiency among healthy infants and toddlers. Arch Pediatr Adolesc Med.

[14] Rajakumar K, Fernstrom JD, Janosky JE, Greenspan SL (2005). Vitamin D insufficiency in preadolescent African-American children. Clin Pediatr (Phila).

[15] Gordon CM, DePeter KC, Feldman HA, Grace E, Emans SJ (2004). Prevalence of vitamin D deficiency among healthy adolescents. Arch Pediatr Adolesc Med.

[16] Lawson M, Thomas M (1999). Vitamin D concentrations in Asian children aged 2 years living in England: population survey. BMJ.

[17] Guillemant J, Le HT, Maria A, Allemandou A, Peres G, Guillemant S (2001). Wintertime vitamin D deficiency in male adolescents: effect on parathyroid function and response to vitamin D3 supplements. Osteoporos Int.

[18] Nicolaidou P, Hatzistamatiou Z, Papadopoulou A, Kaleyias J, Floropoulou E, Lagona E, Tsagris V, Costalos C, Antsaklis A (2006). Low vitamin D status in mother-newborn pairs in Greece. Calcif Tissue Int.

[19] El-Hajj Fuleihan G, Nabulsi M, Choucair M, Salamoun M, Hajj Shahine C, Kizirian A, Tannous R (2001). Hypovitaminosis D in healthy schoolchildren. Pediatrics.

[20] Pehlivan I, Hatun S, Aydogan M, Babaoglu K, Gokalp AS (2003). Maternal vitamin D deficiency and vitamin D supplementation in healthy infants. Turk J Pediatr.

[21] Du X, Greenfield H, Fraser DR, Ge K, Trube A, Wang Y (2001). Vitamin D deficiency and associated factors in adolescent girls in Beijing. Am J Clin Nutr.

[22] Lehtonen-Veromaa M, Mottonen T, Irjala K, Karkkainen M, Lamberg-Allardt C, Hakola P, Viikari J (1999). Vitamin D intake is low and hypovitaminosis D common in healthy 9- to 15-year-old Finnish girls. Eur J Clin Nutr.

[23] Outila TA, Kärkkainen MU, Lamberg-Allardt CJ (2001). Vitamin D status affects serum parathyroid hormone concentrations during winter in female adolescents: associations with forearm bone mineral density. Am J Clin Nutr.

[24] Ward LM, Gaboury I, Ladhani M, Zlotkin S (2007). Vitamin D-deficiency rickets among children in Canada. CMAJ.

[25] Franchi B, Piazza M, Sandri M, Tenero L, Comberiati P, Boner AL, Capristo C (2015). 25-hydroxyvitamin D serum level in children of different ethnicity living in Italy. Eur J Pediatr.

[26] Chiappini E, Vierucci F, Ghetti F, de Martino M, Galli L (2016). Vitamin D status and predictors of Hypovitaminosis D in internationally adopted children. PLoS One.

[27] Shaw NJ, Mughal MZ (2013). Vitamin D and child health: part 2 (extraskeletal and other aspects). Arch Dis Child.

[28] Holick MF, Binkley NC, Bischoff-Ferrari HA, Gordon CM, Hanley DA, Heaney RP, Murad MH, Weaver CM (2011). Evaluation, treatment, and prevention of vitamin D deficiency: an Endocrine Society clinical practice guideline. J Clin Endocrinol Metab.

[29] Holick MF, Binkley NC, Bischoff-Ferrari HA, Gordon CM, Hanley DA, Heaney RP, Murad MH, Weaver CM (2012). Guidelines for preventing and treating vitamin D deficiency and insufficiency revisited. J Clin Endocrinol Metab.

[30] Commissione per le Adozioni Internazionali della Presidenza del Consiglio dei Ministri. Dati e prospettive nelle Adozioni Internazionali. CAI. 2017:1–120. Accessed 11 March 2018 http://www.commissioneadozioni.it/media/153043/report_statistico_2014-2015.pdf

[31] Nikooyeh B, Abdollahi Z, Hajifaraji M, Alavi-Majd H, Salehi F, Yarparvar AH, Neyestani TR (2017). Vitamin D status, latitude and their associations with some health parameters in children: National Food and nutrition surveillance. J Trop Pediatr.

[32] Gilbert-Diamond D, Baylin A, Mora-Plazas M, Marin C, Arsenault JE, Hughes MD, Willett WC, Villamor E (2010). Vitamin D deficiency and anthropometric indicators of adiposity in school-age children: a prospective study. Am J Clin Nutr.

[33] Wortsman J, Matsuoka LY, Chen TC, Lu Z, Holick MF (2000). Decreased bioavailability of vitamin D in obesity. Am J Clin Nutr.

[34] Arunabh S, Pollack S, Yeh J, Aloia JF (2003). Body fat content and 25-hydroxyvitamin D levels in healthy women. J Clin Endocrinol Metab.

[35] Hintzpeter B, Scheidt-Nave C, Muller MJ, Schenk L, Mensink GBM (2008). Higher prevalence of vitamin D deficiency is associated with immigrant background among children and adolescents in Germany. J Nutr.

[36] Lagunova Z, Porojnicu AC, Lindberg F, Hexeberg S, Moan J (2009). The dependency of vitamin D status on body mass index, gender, age and season. Anticancer Res.

[37] Hagenau T, Vest R, Gissel TN, Poulsen CS, Erlandsen M, Mosekilde L, Vestergaard P (2009). Global vitamin D levels in relation to age, gender, skin pigmentation and latitude: an ecologic meta-regression analysis. Osteoporos Int.

[38] Talaat IM, Kamal NM, Alghamdi HA, Alharthi AA, Alshahrani MA (2016). A randomized clinical trial comparing 3 different replacement regimens of vitamin D in clinically asymptomatic pediatrics and adolescents with vitamin D insufficiency. Ital J Pediatr.

[39] Vierucci F, Del Pistoia M, Fanos M, Gori M, Carlone G, Erba P, Massimetti G, Federico G, Saggese G (2013). Vitamin D status and predictors of hypovitaminosis D in Italian children and adolescents: a cross-sectional study. Eur J Pediatr.

[40] Gustafson KL, Eckerle JK, Howard CR, Andrews B, Polgreen LE (2013). Prevalence of vitamin D deficiency in international adoptees within the first 6 months after adoption. Clin Pediatr (Phila).

[41] Razzaghy-Azar M, Shakiba M (2010). Assessment of vitamin D status in healthy children and adolescents living in Tehran and its relation to iPTH, gender, weight and height. Ann Hum Biol.

[42] Kimlin MG (2004). The climatology of vitamin D producing ultraviolet radiation over the United States. J Steroid Biochem Mol Biol.

[43] Webb AR, Kline L, Holick MF (1988). Influence of season and latitude on the cutaneous synthesis of vitamin D3: exposure to winter sunlight in Boston and Edmonton will not promote vitamin D3 synthesis in human skin. J Clin Endocrinol Metab.

[44] Bustos BR, Rodriguez-Nunez I, Pena Zavala R, Soto Germani G (2016). Vitamin D deficiency in children admitted to the paediatric intensive care unit. Rev Chil Pediatr.

[45] Geng S-S, Ma J-Q, Liu S-S, Zhang J, Sheng X-Y (2016). Vitamin D Insufficiency and Its Association with Biochemical and Anthropometric Variables of Young Children in Rural Southwestern China. Chin Med J.

[46] Kapil U, Pandey RM, Goswami R, Sharma B, Sharma N, Ramakrishnan L, Singh G, Sareen N, Sati HC, Gupta A, Sofi NY (2017). Prevalence of vitamin D deficiency and associated risk factors among children residing at high altitude in Shimla district, Himachal Pradesh, India. Indian J Endocrinol Metab.

[47] Chen TC, Chimeh F, Lu Z, Mathieu J, Person KS, Zhang A, Kohn N, Martinello S, Berkowitz R, Holick MF (2007). Factors that influence the cutaneous synthesis and dietary sources of vitamin D. Arch Biochem Biophys.

[48] Cheng S, Tylavsky F, Kroger H, Karkkainen M, Lyytikainen A, Koistinen A, Mahonen A, Alen M, Halleen J, Vaananen K, Lamberg-Allardt C (2003). Association of low 25-hydroxyvitamin D concentrations with elevated parathyroid hormone concentrations and low cortical bone density in early pubertal and prepubertal Finnish girls. Am J Clin Nutr.

[49] Bellone S, Esposito S, Giglione E, Genoni G, Fiorito C, Petri A, Bona G, Prodam F (2014). Vitamin D levels in a paediatric population of normal weight and obese subjects. J Endocrinol Investig.

[50] Cadario F, Savastio S, Magnani C, Cena T, Pagliardini V, Bellomo G, Bagnati M, Vidali M, Pozzi E, Pamparana S, Zaffaroni M, Genoni G, Bona G (2015). High prevalence of vitamin D deficiency in native versus migrant mothers and newborns in the north of Italy: a call to act with a stronger prevention program. PLoS One.

[51] Turney K, Wildeman C. Mental and physical health of children in Foster Care. Pediatrics. 2016; 10.1542/peds.2016-1118. 10.1542/peds.2016-1118 27940775

[52] Hobbs GF, Hobbs CJ, Wynne JM (1999). Abuse of children in foster and residential care. Child Abuse Negl.

[53] Ferrara P, Romani L, Bottaro G, Ianniello F, Fabrizio GC, Chiaretti A, Alvaro F (2013). The physical and mental health of children in foster care. Iran J Public Health.

[54] Vierucci F, Del Pistoia M, Fanos M, Erba P, Saggese G (2014). Prevalence of hypovitaminosis D and predictors of vitamin D status in Italian healthy adolescents. Ital J Pediatr.

[55] Erkal MZ, Wilde J, Bilgin Y, Akinci A, Demir E, Bodeker RH, Mann M, Bretzel RG, Stracke H, Holick MF (2006). High prevalence of vitamin D deficiency, secondary hyperparathyroidism and generalized bone pain in Turkish immigrants in Germany: identification of risk factors. Osteoporos Int.

[56] Juttmann JR, Visser TJ, Buurman C, de Kam E, Birkenhager JC (1981). Seasonal fluctuations in serum concentrations of vitamin D metabolites in normal subjects. Br Med J (Clin Res Ed).

[57] Tjellesen L, Christiansen C (1983). Vitamin D metabolites in normal subjects during one year. A longitudinal study. Scand J Clin Lab Invest.

[58] Hegarty V, Woodhouse P, Khaw KT (1994). Seasonal variation in 25-hydroxyvitamin D and parathyroid hormone concentrations in healthy elderly people. Age Ageing.

[59] Scharla SH, Scheidt-Nave C, Leidig G, Woitge H, Wuster C, Seibel MJ, Ziegler R (1996). Lower serum 25-hydroxyvitamin D is associated with increased bone resorption markers and lower bone density at the proximal femur in normal females: a population-based study. Exp Clin Endocrinol Diabetes.

[60] Woitge HW, Scheidt-Nave C, Kissling C, Leidig-Bruckner G, Meyer K, Grauer A, Scharla SH, Ziegler R, Seibel MJ (1998). Seasonal variation of biochemical indexes of bone turnover: results of a population-based study. J Clin Endocrinol Metab.

[61] Krall EA, Sahyoun N, Tannenbaum S, Dallal GE, Dawson-Hughes B (1989). Effect of vitamin D intake on seasonal variations in parathyroid hormone secretion in postmenopausal women. New Engl J Med.

[62] Chapuy MC, Schott AM, Garnero P, Hans D, Delmas PD, Meunier PJ (1996). Healthy elderly French women living at home have secondary hyperparathyroidism and high bone turnover in winter. EPIDOS Study Group. J Clin Endocrinol Metab.

[63] Brot C, Vestergaard P, Kolthoff N, Gram J, Hermann AP, Sorensen OH (2001). Vitamin D status and its adequacy in healthy Danish perimenopausal women: relationships to dietary intake, sun exposure and serum parathyroid hormone. Br J Nutr.

[64] Marwaha RK, Tandon N, Reddy DRHK, Aggarwal R, Singh R, Sawhney RC, Saluja B, Ganie MA, Singh S (2005). Vitamin D and bone mineral density status of healthy schoolchildren in northern India. Am J Clin Nutr.

[65] Steingrimsdottir L, Gunnarsson O, Indridason OS, Franzson L, Sigurdsson G (2005). Relationship between serum parathyroid hormone levels, vitamin D sufficiency, and calcium intake. JAMA.

[66] Aggarwal V, Seth A, Aneja S, Sharma B, Sonkar P, Singh S, Marwaha RK (2012). Role of calcium deficiency in development of nutritional rickets in Indian children: a case control study. J Clin Endocrinol Metab.

[67] DeLucia MC, Mitnick ME, Carpenter TO (2003). Nutritional rickets with normal circulating 25-hydroxyvitamin D: a call for reexamining the role of dietary calcium intake in north American infants. J Clin Endocrinol Metab.

