# Supplementary material for: Epidemiology and risk factors of hypovitaminosis D in a cohort of internationally adopted children: a retrospective study
Source: Ital J Pediatr. 2018 Jul 27;44:86. doi: 10.1186/s13052-018-0527-4 (PMC6062984; doi:10.1186/s13052-018-0527-4)
Supplement: Supplementary file 1 — Post hoc analyses. (DOCX 17 kb) [file 13052_2018_527_MOESM1_ESM.docx]

**Supplementary data – Post hoc analyses**

|  | **Vitamin D status** | | | | | | **Bonferroni-adjusted p-value for significance** |
| --- | --- | --- | --- | --- | --- | --- | --- |
|  | ***Severe VDD vs Moderate VDD*** | ***Severe VDD vs Mild VDD*** | ***Severe VDD vs normal VD*** | ***Moderate VDD vs Mild VDD*** | ***Moderate VDD vs Normal VD*** | ***Mild VDD vs Normal VD*** |  |
| ***Sex*** | **0.007** | 0.172 | 0.068 | 0.103 | 0.599 | 0.519 | 0.017 |
|  |  |  |  |  |  |  |  |
| ***Skin color*** |  |  |  |  |  |  | 0.003 |
| *Very fair/fair vs Intermediate* | 0.155 | 0.084 | 0.017 | 0.640 | 0.160 | 0.339 |  |
| *Very fair/fair vs Olive/Brown* | 0.244 | 0.008 | 0.018 | 0.044 | 0.085 | 0.877 |  |
| *Intermediate vs Olive/Brown* | 0.010 | **< 0.0001** | **< 0.0001** | 0.008 | **0.001** | 0.317 |  |
|  |  |  |  |  |  |  |  |
| ***Macro-area*** |  |  |  |  |  |  | 0.001 |
| *Europe vs Latin America* | 0.638 | 0.129 | 0.327 | 0.177 | 0.425 | 0.864 |  |
| *Europe vs Asia* | 0.274 | 0.408 | 0.092 | 0.015 | 0.002 | 0.321 |  |
| *Europe vs Africa* | 0.036 | 0.010 | 0.275 | 0.389 | 0.601 | 0.266 |  |
| *Latin America vs Asia* | 0.791 | 0.462 | 0.622 | 0.221 | 0.008 | 0.140 |  |
| *Latin America vs Africa* | 0.081 | 0.192 | 0.845 | 0.534 | 0.110 | 0.292 |  |
| *Asia vs Africa* | **0.0007** | 0.037 | 0.850 | 0.074 | **< 0.0001** | 0.010 |  |
|  |  |  |  |  |  |  |  |
| ***Climatic zone*** |  |  |  |  |  |  | 0.003 |
| *Temperate vs subtropical* | 0.106 | 0.068 | 0.004 | 0.726 | 0.063 | 0.115 |  |
| *Temperate vs tropical* | 0.346 | 0.494 | 0.162 | 0.781 | 0.420 | 0.285 |  |
| *Subtropical vs tropical* | 0.007 | 0.009 | **< 0.0001** | > 0.9999 | 0.006 | 0.010 |  |
|  |  |  |  |  |  |  |  |
| ***Housing solution*** |  |  |  |  |  |  | 0.003 |
| *Foster home vs foster family* | 0.247 | 0.762 | 0.132 | 0.296 | **0.0005** | 0.026 |  |
| *Foster home vs orphanage* | > 0.9999 | > 0.9999 | 0.101 | > 0.9999 | 0.021 | 0.027 |  |
| *Foster family vs orphanage* | 0.132 | 0.818 | 0.524 | 0.086 | 0.014 | 0.241 |  |
|  |  |  |  |  |  |  |  |
| ***Season at first blood draw*** |  |  |  |  |  |  | 0.001 |
| *Spring vs summer* | 0.014 | **< 0.0001** | **< 0.0001** | **0.0006** | **< 0.0001** | 0.010 |  |
| *Spring vs fall* | 0.106 | **0.0006** | 0.005 | 0.004 | 0.047 | > 0.9999 |  |
| *Spring vs winter* | 0.069 | 0.244 | 0.335 | 0.643 | 0.865 | > 0.9999 |  |
| *Summer vs fall* | 0.196 | 0.081 | **0.0005** | 0.267 | **< 0.0001** | **0.001** |  |
| *Summer vs winter* | **0.0003** | **< 0.0001** | **< 0.0001** | 0.002 | **< 0.0001** | 0.005 |  |
| *Fall vs winter* | **0.0004** | **< 0.0001** | **< 0.0001** | 0.013 | 0.082 | > 0.99990 |  |
|  |  |  |  |  |  |  |  |
| ***ALP Status*** | 0.038 | **0.003** | > 0.9999 | 0.225 | 0.023 | **0.001** | 0.008 |
|  |  |  |  |  |  |  |  |
| ***P Status*** |  |  |  |  |  |  | 0.003 |
| *Normal vs decreased* | > 0.9999 | 0.482 | 0.398 | 0.199 | 0.352 | 0.05 |  |
| *Normal vs increased* | 0.079 | 0.013 | 0.013 | 0.272 | 0.219 | 0.765 |  |
| *Decreased vs Increased* | 0.228 | 0.477 | 0.020 | 0.776 | 0.098 | 0.072 |  |
